# Human Epidermal Growth Factor 2 (Her-2) Expression in Gastric and Gastroesophageal Carcinomas: A Clinicopathological Evaluation in a Tertiary Care Institute

**DOI:** 10.7759/cureus.67895

**Published:** 2024-08-27

**Authors:** Rizwana Shaik, Inuganti Venkata Renuka, Sudhakar Ramamoorthy, Ramya Potti, Lakshmi Pranathi

**Affiliations:** 1 Pathology, NRI Medical College, Guntur, IND; 2 Pathology, NRI Medical College, Guntur , IND

**Keywords:** gastroesophageal, gastric carcinoma, immunohistochemistry, her-2, lauren classification

## Abstract

Introduction

Gastric carcinoma is a significant global health concern, known for its high mortality rate. HER-2 overexpression is observed in a notable proportion of gastric carcinoma and is associated with a worse prognosis. However, HER-2 expression enables targeting the protein by monoclonal antibodies that improve overall survival in HER-2-positive gastric cancers. This study aims to evaluate the HER-2 expression in gastric and gastroesophageal carcinomas.

Materials and methods

This observational study was conducted in the Department of Pathology, involving 60 endoscopic biopsy and resection specimens of gastric and gastroesophageal carcinomas. HER-2 expression was assessed by immunohistochemistry (IHC) based on the Trastuzumab for GAstric Cancer (ToGA) trial’s scoring system. The primary outcome was HER-2 status, with statistical analysis performed to evaluate associations with various clinicopathological parameters.

Results

Among 60 cases, 26 (43.3%) showed HER-2 positivity. HER-2 positivity was significantly (p=0.004) associated with age, being higher in 20-39 years and ≥80 years age groups. Gender and tumor location were not significantly associated with HER-2 positivity. Moderate and poorly differentiated carcinomas exhibited higher HER-2 positivity. Histological types, tubular adenocarcinoma, and papillary adenocarcinoma showed significant (p=0.01) association with HER-2 positivity compared to other types.

Conclusion

HER-2 status assessment is crucial in managing gastric and gastroesophageal carcinomas. HER-2 positivity is notably higher in certain age groups and histological types particularly tubular and papillary adenocarcinoma, and in moderately to poorly differentiated carcinomas. These insights can aid in selecting appropriate gastric and gastroesophageal carcinomas that warrant HER-2 testing on IHC. Identifying gastric and gastroesophageal carcinomas that show HER2 expression may highlight potential candidates for targeted therapy.

## Introduction

Gastric carcinoma is a prevalent cancer type and a leading cause of cancer-related death globally. As reported by GLOBOCON 2020, it ranked as the fifth most common cancer and the fourth leading cause of cancer-related mortality worldwide [1]. In India, gastric carcinoma stands out as the most common gastrointestinal cancer, accounting for 9% of cases. It is the third most common cancer site among females and the second among males [2].

Human epidermal growth factor receptor 2 (HER-2) protein encoded by the *ERBB2 *gene, is a key driver of tumorigenesis and HER-2 overexpression as a result of *ERBB2* (HER-2) gene amplification has been observed in several other solid tumors also [3]. HER-2 is overexpressed in 7%-34% of gastric tumors [4]. HER-2 amplification in these tumors is associated with poor prognosis when compared with HER-2 non-amplified tumors [5]. Trastuzumab is a biological therapeutic agent and a monoclonal antibody that targets the extracellular domain of the HER-2 receptor on positive cancer cells [6]. It has now been tried in the treatment of gastric carcinoma and has been shown to increase survival in gastric cancers when HER-2 is overexpressed. More than 20% of gastric cancers now are found to show overexpression of HER-2 and this percentage increases to 33% in gastroesophageal junction tumors [7].

The aim of this study is to evaluate HER-2 expression in gastric and gastroesophageal carcinomas and investigate its association with various clinicopathological parameters. The objectives are to determine the prevalence of HER-2 positivity in these cases and analyze how HER-2 positivity correlates with clinicopathological parameters.

## Materials and methods

The study was conducted in the Department of Pathology for histopathological evaluation and immunohistochemical (IHC) correlation. This is an observational hospital-based study with 60 subjects who were diagnosed with gastric or gastroesophageal carcinomas on endoscopic biopsy (34 cases) or resection specimen (26 cases). Where both biopsy and resection specimens were available for the same patient, the findings of the resection specimen were considered for analysis. The study was approved by our hospital's institutional ethics committee (NRIAS/IEC/215/2015). Biopsy-proven gastric or gastroesophageal carcinomas of varied histological types were subjected to IHC staining for HER-2 using the HercepTest kit (Dako; Agilent, USA). The procedure begins with deparaffinization and rehydration of formalin-fixed, paraffin-embedded tissue sections with 4-micron thickness. Antigen retrieval is then performed to unmask HER-2 epitopes, followed by endogenous blocking using peroxidase for 5 minutes. The tissue is then incubated for 30 minutes with primary antibody specific to HER-2 and detection is achieved using the application of horse radish peroxidase for 30 minutes and chromogenic substrate for 10 minutes. Finally, the slides were counterstained with hematoxylin and HER-2 expression was evaluated based on the intensity and percentage of membrane staining in tumor cells. Cases with equivocal HER-2 IHC score (score 2) lacking subsequent HER-2 fluorescent in-situ hybridization testing were excluded from the study. The Trastuzumab for GAstric Cancer (ToGA) trial’s scoring system [8] was applied, assessing the HER-2 membranous expression pattern as described in Table 1.

**Table 1:** HER-2 IHC scoring criteria based on ToGA trial HER-2 - Human epidermal growth factor 2, IHC - immunohistochemistry, ToGA - Trastuzumab for GAstric Cancer *Cancer cell cluster consisting of ≥5 neoplastic cells

HER-2 IHC score	HER-2 IHC pattern in surgical specimen	HER-2 IHC pattern in biopsy	HER-2 expression assessment
0	No reactivity or membranous reactivity in <10% tumor cells	No reactivity or membranous reactivity in any cancer cell	Negative
1+	Faint or barely perceptible membranous reactivity in ≥10% of cancer cells	Cancer cell cluster* with a faint or barely perceptible membranous reactivity irrespective of percentage of cancer cells positive	Negative
2+	Weak or moderate basolateral or lateral membranous reactivity in ≥10% of cancer cells	Cancer cell cluster* with a weak to moderate complete, basolateral or lateral membranous reactivity irrespective of percentage of cancer cells positive	Equivocal
3+	Strong complete, basolateral or lateral membranous reactivity in ≥10% of cancer cells	Cancer cell cluster* with a strong complete, basolateral or lateral membranous reactivity irrespective of percentage of cancer cells positive	Positive

HER-2 status was the primary outcome variable. Histologic types and tumor differentiation were noted along with socio-demographic variables like age and gender. Descriptive analysis of the data was done by frequency and percentages for categorical variables and mean and standard deviation for quantitative variables. The chi-square test was used to test the statistical significance of the association, with a p-value ≤ 0.05 was considered statistically significant.

## Results

A total of 60 cases were included in the study of which 26 (43.3%) showed HER-2 positivity and 34 cases (56.7%) were negative. Table 2 details HER-2 positivity across various clinicopathological parameters.

**Table 2:** Clinicopathological characteristics of gastric and gastroesophageal junction carcinomas and their association with HER-2 expression % - Percentage of HER-2 positivity, X2 - Chi square value, HER-2 - Human epidermal growth factor 2 p-value ≤ 0.05 is considered significant

Clinicopathological characteristics		Number of cases	Number of HER-2 positive cases (%)	X^2^	P-value
Total study subjects		60	26 (43.3)		
Age	20-39 years	3	3 (100)	13.18	0.004
	40-59 years	25	7 (28)		
	60-79 years	28	13 (46.4)		
	≥80 years	4	3 (75)		
Gender	Males	41	19 (46.3)	0.47	0.49
	Females	19	7 (36.8)		
Tumor site	Gastric	50	21 (42)	0.29	0.59
	Gastroesophageal junction	10	5 (50)		
Histologic grade	Well differentiated	32	11 (34.4)	5.83	0.05
	Moderately differentiated	10	6 (60)		
	Poorly differentiated	18	9 (50)		
Histologic type (WHO Classification)	Poorly cohesive carcinoma	10	3 (30)	10.85	0.01
	Papillary adenocarcinoma	4	2 (50)		
	Mucinous adenocarcinoma	8	0		
	Tubular adenocarcinoma	38	21 (55.2)		

**Figure 1:**
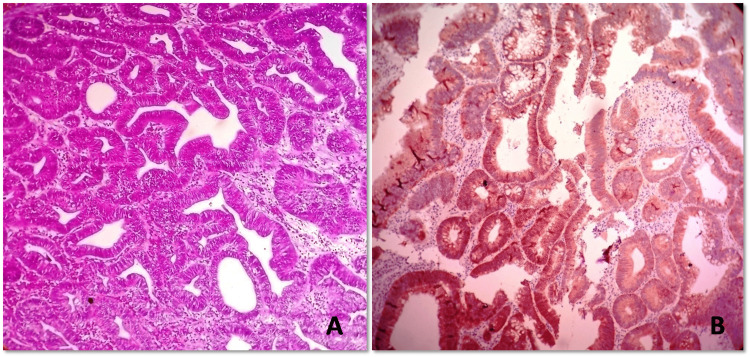
Histomorphology and HER-2 expression pattern of tubular adenocarcinoma (A) Low power magnification of gastric tubular adenocarcinoma shows tubules lined by columnar tumor cells with nuclear stratification and coarse chromatin. Surrounding stroma is desmoplastic (hematoxylin & eosin, 200x). (B) HER-2 immunohistochemistry shows basolateral membranous positivity in more than 10% tumor cells (score 3) (HER-2 IHC, 200x). HER-2 - Human epidermal growth factor 2, IHC - immunohistochemistry

**Figure 2:**
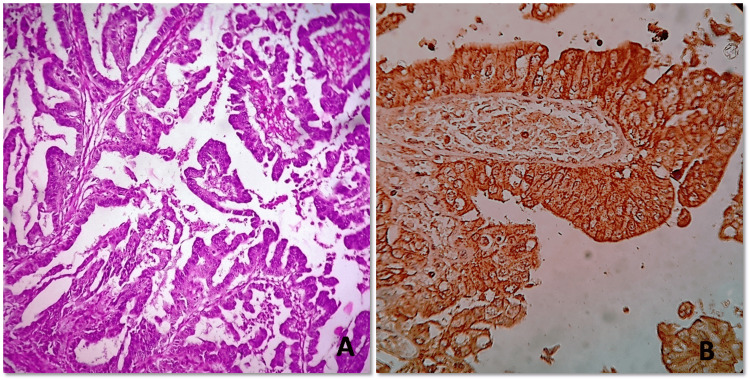
Histomorphology and HER-2 expression pattern of gastric papillary adenocarcinoma (A) Low power magnification of gastric papillary adenocarcinoma shows papillae lined by columnar tumor cells with nuclear stratification, coarse chromatin and prominent nucleoli (Hematoxylin & eosin, 200x). (B) HER-2 immunohistochemistry shows membranous positivity in more than 10% of tumor cells (score 3) (HER-2 IHC, 400x). HER-2 - Human epidermal growth factor 2, IHC - immunohistochemistry

The association between age and HER-2 positivity was statistically significant and HER-2 positivity was noted in three out of three (100%) of 20-39 years age group and three out of four (75%) ≥80 years age group. Among histological grades, significant HER-2 positivity was noted in six out of 10 (60%) moderately differentiated and nine out of 18 (50%) of poorly differentiated tumor cases. Among four different histological types (based on WHO classification) documented in our study, HER-2 positivity cases were higher in patients with tubular adenocarcinoma and papillary adenocarcinoma. Figures 1A, 1B, 2A, 2B illustrate histomorphology and membranous positivity of HER-2 immunohistochemistry (IHC). No significant association with HER-2 positivity was achieved for gender and tumor site. Spearman's rank correlation between HER-2 positivity and tumor grade showed a positive correlation (Spearman's rho=0.32, p=0.02) indicating that higher tumor grades tend to have higher HER-2 positivity. Combining papillary and tubular adenocarcinoma as predictors, the positive predictive value (PPV) for HER-2 positivity was calculated to be 88%, indicating a high probability that HER-2 positive cases correspond to these histological subtypes.

## Discussion

Gastric carcinoma is a major public health concern, being the second leading cause of cancer death. The five-year survival for advanced disease is below 15% despite treatment [1]. Recent research focuses on molecularly targeted agents like HER-2. HER-2 expression rates in gastric carcinoma range from 4% to 44% on IHC [9]. In our study, we observed HER-2 expression in 43.3% of gastric and gastroesophageal carcinomas, aligning closely with several reports from the Indian subcontinent. For instance, Sekharan et al. [10] documented an HER-2 positivity rate of 44.2%, while Tewari et al [11] and Rajagopal et al [12] reported HER-2 expression rates of 21.4% and 26.7% respectively. Over 20% of gastric carcinomas now have shown HER-2 overexpression, increasing to 33% in gastroesophageal junction tumors [13]. In our study, HER-2 positivity was found in 42% of gastric carcinomas and 50% of gastroesophageal tumors. The median age identified in our study was 58 years with a range between 26 and 89 years of age. The age incidence was in concordance with other studies [11,12,14-16] listed in Table 3.

**Table 3:** Median age group of gastric and gastroesophageal carcinoma cases reported in the literature

Research studies	Year of study	No of cases	Median age in years (range)
Tewari et al [11]	2015	70	52.7(30-71)
Rajagopal et al [12]	2015	60	65.65
U prak et al [14]	2015	135	61(29-84)
Kataoka et al [15]	2013	213	71.2
Shan et al [16]	2013	2013	58(20-82)
Present study	2024	60	58(26-89)

HER-2 positive cases were significantly high in extremes of age, contrasting slightly with findings by Yanfeng et al. [17], where the authors detected a progressive increase in HER-2 positive cases as age increases. Gender and tumor location did not have any association with HER-2 positivity as mentioned in the study by Li et al. [18]. HER-2 positivity in different histologic grades was 34% (11 out of 32 cases), 60% (six out of 10 cases), and 50% (nine out of 18 cases) in well-differentiated, moderately differentiated, and poorly differentiated respectively. There was a trend towards HER-2 positivity in poorly differentiated tumors which underscores the potential role of HER2 as a marker for aggressive tumor behavior. However, the current study is in contrast to Oono et al. [19], where differentiated histology carries 81% HER-2 positivity against undifferentiated tumors (19%). Among histologic types based on WHO classification, tubular and papillary adenocarcinoma showed significantly higher HER-2 positive cases than poorly cohesive and mucinous carcinoma. These findings correlate with previous studies where tubular adenocarcinoma (95%) [20] and papillary adenocarcinoma (62%) [17] showed higher HER-2 expression. Similarly, Ieni et al. [21] showed higher HER-2 expression among tubular and papillary adenocarcinoma compared to other histologic types. A critical challenge in assessing HER-2 status is the inherent tumor heterogeneity observed in gastric and gastroesophageal carcinomas. A study of large sample size showed HER-2 heterogeneity in 50% of cases when IHC was performed in a single block and 30.10% of cases when performed in two representative blocks [22]. In the current study, IHC was performed in a single block per case, thus posing a limitation to interpreting HER-2 heterogeneity in tumors. Limited sample size and exclusion of equivocal HER-2 scores that lack HER-2 FISH findings were also the limitations of the current study. Studies on a larger scale through multi-institutional study or meta-analysis are warranted for better correlation and developing treatment regimens against HER-2 in gastric and gastroesophageal carcinoma cases.

## Conclusions

This study highlights the association of HER-2 status in gastric and gastroesophageal junction carcinomas with specific clinicopathological features. Our findings indicate that HER-2 positivity is notably higher in patients at the extremes of age, as well as those with tubular adenocarcinoma and papillary adenocarcinoma, and moderately to poorly differentiated tumors. These observations can help identify which cases are more likely to be HER-2 positive and should be prioritized for HER-2 IHC. This approach can assist in selecting appropriate targeted therapies to improve outcomes in HER-2-positive gastric carcinoma cases.
